# An Analysis of Child Abuse Detected by Skeletal Surveys Before and During the COVID‐19 Pandemic

**DOI:** 10.1002/pdi3.2526

**Published:** 2025-02-26

**Authors:** Vincent R. Li, Trevor A. Pickering, Karen Kay Imagawa, Joseph M. Rich, Amit S. Sura

**Affiliations:** ^1^ Department of Radiology Children's Hospital of Los Angeles Los Angeles California USA; ^2^ School of Medicine and Public Health University of Wisconsin Madison Wisconsin USA; ^3^ Department of Population and Public Health Sciences Keck School of Medicine of the University of Southern California Los Angeles California USA; ^4^ Department of Pediatrics Children's Hospital Los Angeles Los Angeles California USA; ^5^ Department of Radiology Keck School of Medicine of the University of Southern California Los Angeles California USA; ^6^ Department of Biology and Biological Engineering California Institute of Technology Pasadena California USA

**Keywords:** child abuse, COVID‐19, radiology

## Abstract

The COVID‐19 pandemic and the stay‐at‐home order may have impacted the risk of child physical abuse (CPA). Analyzing injury types in suspected CPA cases before and during the pandemic can provide valuable insights. The objective of this study is to compare the incidences and types of injuries detected by skeletal surveys in suspected CPA cases at a large academic children's hospital before and during the COVID‐19 pandemic. The study compared the incidences and proportion of specific injury sites, types of traumas, and demographic characteristics before and during the pandemic using Pearson's chi‐squared and Fisher's exact tests. A total of 479 patients who underwent skeletal surveys were included, with 306 patients sampled before the pandemic (63.8%) and 173 patients sampled during the pandemic (36.1%). There were no significant differences in age, gender, or ethnicity between the two groups. Highly suspicious CPA incidences were similar before (0.87/month) and during (0.81/month) the pandemic, reflecting 10.5% and 11.6% of all patients who underwent skeletal surveys, respectively. There were no significant demographic differences between the groups. However, the proportion of patients with intracranial or retinal hemorrhage significantly decreased (*p* < 0.05) during the pandemic, with no significant differences in types of skeletal fractures. In conclusion, the overall incidence of CPA remained similar before and during the pandemic. The significant decrease in intracranial and retinal hemorrhages suggests a potential change in injury patterns or reporting. Further research is needed to understand the factors contributing to these findings.

**Clinical Trial Registration:**

This study is a retrospective clinical trial and therefore not subject to clinical trial registration requirements.

## Graphical abstract

This study analyzes the impact of the COVID‐19 pandemic on child physical abuse patterns, revealing a stable overall incidence but a significant decrease in intracranial and retinal hemorrhages detected by skeletal surveys.

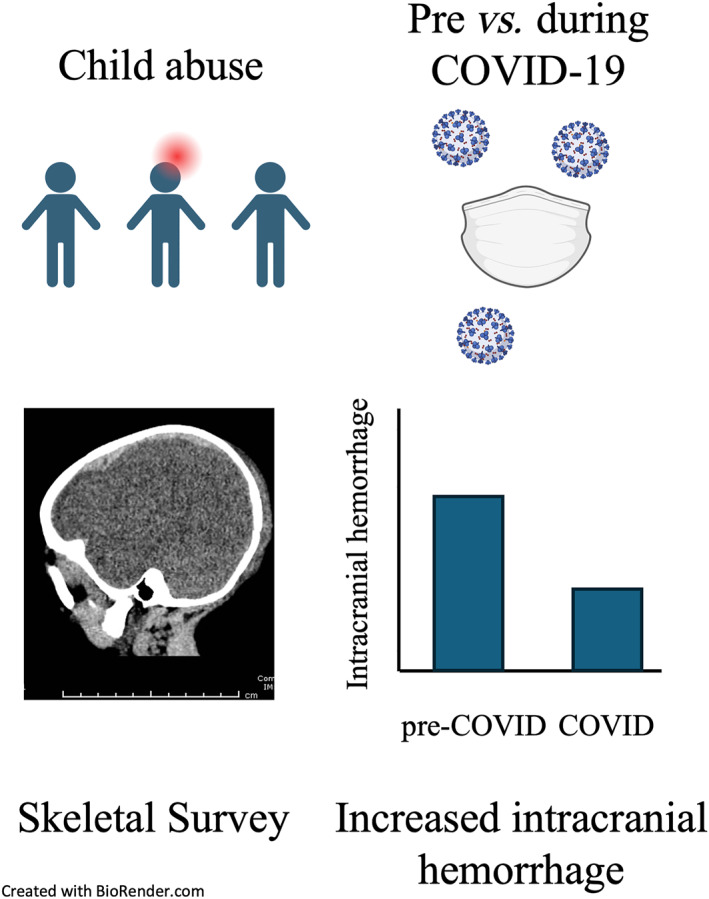

## 1 Introduction

Child physical abuse (CPA) is a leading cause of injury‐related mortality in infants and children, with infants under the age of one at a disproportionate risk [1]. CPA is defined as any deliberate physical harming of a child, which could result from hitting, shaking, burning, pushing, and other physical acts of violence. This does not include neglect, sexual abuse, or psychological abuse, which are other distinct forms of child maltreatment. It is estimated that more than 100,000 children per year in the US are victims of CPA [2]. The causes of CPA have been linked to parental stress such as epidemics or recessions [3]. Injuries with the highest prevalence of CPA include unexplained bruising, fractures, retinal hemorrhages, and intracranial hemorrhages [4, 5]. Patients with multiple suspicious types of injuries occurring simultaneously, or injuries of different ages, are even more specific for CPA [5, 6]. The severity of CPA‐related injuries varies as well, with injuries, such as retinal hemorrhages, being more common in fatal afflictions [4]. With respect to head trauma, neuroradiological findings that influence the degree of CPA likelihood include the location of subdural hemorrhages (i.e., within the interhemispheric fissure, in the posterior fossa, and over the convexity), and the presence of cerebral edema and hypoxic–ischemic injury, all of which have been shown to be significantly associated with abusive head trauma; as opposed to epidural hemorrhage, which is significantly associated with nonabusive head trauma, and subarachnoid hemorrhages and focal parenchymal hemorrhages, which are not discriminatory findings [7, 8, 9].

The COVID‐19 pandemic has influenced many aspects of everyday life, while also being responsible for over 1 million deaths in the United States at the time of this writing [10]. There have been societal modifications in response to the pandemic, with lockdowns or stay‐at‐home orders placed upon cities or countries to lower the infection and mortality rates. The lockdowns and stay‐at‐home orders, although deemed necessary for controlling airborne disease, have had unintentional consequences regarding the economic and emotional well‐being of millions of American citizens. Heightened stress, school closures, loss of income, reduced health service availability, and/or social isolation resulting from the COVID‐19 pandemic is hypothesized to increase the risk for CPA [11]. The Center for Disease Control and Prevention (CDC) has reported that, compared with 2019, the total number of emergency department (ED) visits related to child abuse and neglect (CAN) decreased in 2020 but the percentage of such visits resulting in hospitalization increased during the COVID‐19 pandemic [12]. Similarly, Bullinger et al. reported that the proportion of CAN visits per pediatric ED visits increased by 97%, even though both the overall numbers of pediatric ED visits and CAN‐related ED visits declined [13]. Kovler et al. observed that, compared to the prepandemic, there was an almost 50% increase in CPA injuries at their level I pediatric trauma center during the COVID‐19 pandemic [14]. Taubman‐Ben‐Ari et al. compared stress levels before and during the COVID‐19 pandemic of new parents caring for infants 3–12 months of age and reported that these parents, particularly fathers, had increased parental stress during the pandemic [3]. Such elevated stress could ultimately negatively impact parenting, thus placing young infants' safety and overall health and well‐being at risk.

However, such increases in CAN are not conclusively reported in other studies. Rapp et al. reviewed 12 articles involving CAN inclusive of physical, psychological, and sexual abuse as well as child neglect during the COVID‐19 pandemic, and found that, compared to the prepandemic period, 5 articles presented an increase in CAN, 6 articles reported a decrease, and 1 study found no difference [15]. They reported variations in the geography of the study location, age of CAN victims, and types of CAN assessed. Sege and Stephens reported another review of available data that indicated there was not a significant rise in child abuse related to COVID‐19 [16]. Although there is a multitude of peer‐reviewed articles addressing either an increase, decrease, or constant rate of child abuse before and during the pandemic, there is a noticeable gap in the literature concerning the specific types of injuries sustained during CPA, especially in relation to how these injuries are detected and classified through medical imaging and physical examination. To our knowledge, no study has comprehensively compared the types and sites of CPA injuries detected through skeletal surveys and radiographic imaging before and during the pandemic. Addressing this gap will provide a deeper understanding of how the pandemic may have altered the nature and severity of CPA injuries. The primary objective of this study was twofold: (1) assess and compare the incidence and types of CPA detected via skeletal surveys before and during the pandemic through retrospective review of the medical records of pediatric patients evaluated at a large California academic children's hospital and (2) investigate any statistical differences in the incidences, types, and sites of injuries related to CPA detected from radiographic imaging and/or physical exam between the two time periods.

## 2 Materials and Methods

Following Institutional Rewiew Board (IRB) approval, electronic medical records for all patients who underwent skeletal surveys for potential CPA at a large metropolitan academic children's hospital from January 1, 2019 through December 31, 2020 were retroactively reviewed. All patients were evaluated through a CPA workup, including pertinent radiographic and/or laboratory studies, obtaining any history pertaining to the mechanism(s) of injury provided by the caregivers, clinical assessments by the health care providers and hospital social workers, and/or consultation by the hospital's child protection team.

Patients were classified as having an initial encounter date of either before (before the pandemic) or on or after (during the pandemic) March 19, 2020, when the stay‐at‐home order was first enacted in the state of California. A combination of injury data, clinical documentation, and notes from hospital social workers and/or the child protection team along with discharge summaries were used to determine the likelihood and level of suspicion for CPA. Patients who displayed multiple criteria including foster home placement, multiple CPA‐type injuries, and/or an indication of CPA from documentation were determined to have a high level of suspicion for CPA. For these patients' demographic characteristics and radiographic findings, including fracture type(s), were collected and reviewed. Demographic variables included age, gender, and ethnicity (i.e., African American, Hispanic, or Other). Types of traumas tracked included retinal and cerebral hemorrhages, cerebral infarction, and skeletal fractures, including injuries resulting in death. The proportion of patients with each demographic characteristic and type and site of trauma, as well as incidence of CPA for all patients with a skeletal survey, were compared before vs. during the pandemic using Pearson’s chi‐squared test and Fisher’s exact test, and the exact test for independence of rates was used to compare the proportion and incidences before and during the pandemic.

## 3 Results

A review of records of 479 patients who underwent skeletal surveys indicated that 306/479 (63.9%) patients were surveyed before the pandemic and 173/479 (36.1%) patients were surveyed during the pandemic. For 427/479 (89.1% patients who underwent a skeletal survey, both before and during the pandemic), CPA was either ruled out, suspicion for CPA was low, and/or the findings were deemed inconclusive for accidental versus nonaccidental/inflicted trauma or the skeletal survey was performed for alternative medical reason/diagnoses. The most reported injury mechanisms for infants with a low and/or inconclusive concern for CPA were simple short falls (e.g., off beds, out of strollers, or from the arms of a caregiver), although still frequently led to skeletal fractures and/or small intracranial hemorrhage. One of the patients without suspected nonaccidental trauma sustained retinal hemorrhages, which were deemed most likely to be related to birth trauma.

**FIGURE 1:**
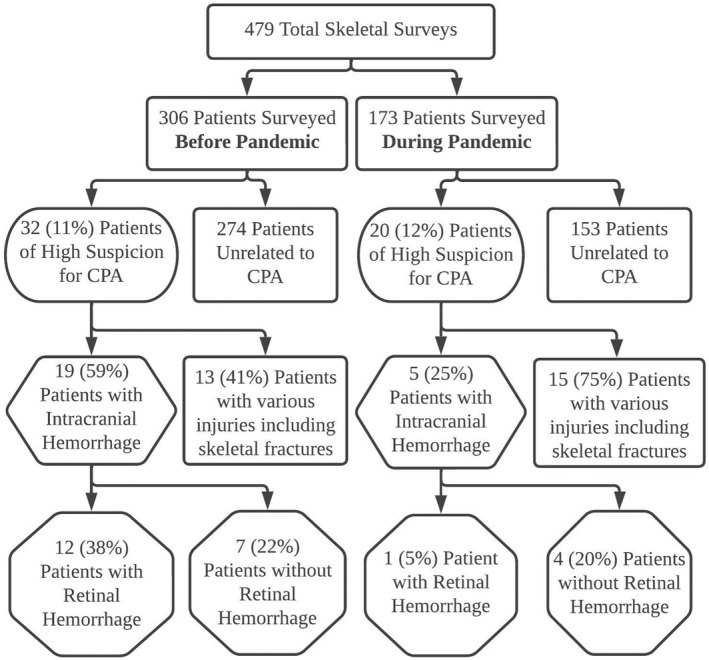
A flowchart of skeletal survey samples and incidences of patients with a high level of suspicion for child physical abuse (CPA) before and during the COVID‐19 pandemic.

Of the patient group surveyed before the pandemic, a high level of suspicion for CPA was noted in 32/306 (10.5%) of the patients (0.87 per month, 95% CI = 0.59–1.23). The patient group surveyed during the pandemic yielded 20/173 (11.6%) of the patients with a high level of suspicion for CPA (0.81 per month, 95% CI = 0.49–1.25). The average monthly incidence of CPA among children with a skeletal survey was not significantly different before versus during the pandemic (*p*  = 0.81). Table 1 compares the demographic and injury characteristics of the patients with a high level of suspicion for CPA identified before and during the pandemic. There were no significant demographic characteristics (i.e., age, gender, and ethnicity) for patients with a high suspicion of CPA before versus during the pandemic (*p* = 0.092; *p* = 0.700; *p* > 0.900, respectively) . The total proportion of CPA with retinal hemorrhages (12/32 [38%] and (1/20 [5%]) or intracranial hemorrhage (19/32 [59%] and (5/20 [25%]) significantly decreased (*p* = 0.008 and 0.016, respectively) during the pandemic. All patients before and during the pandemic with retinal hemorrhages were also accompanied by intracranial hemorrhage. Of these patients before the pandemic, three (25%) patients had accompanying skeletal fracture(s) and three (25%) patients died due to abusive head trauma. Multiple varieties of skeletal fractures, for example, fractures of the skull, rib, and upper and lower extremities as well as clavicles were observed, and their incidences before and during the pandemic were not significantly different. Figure 1 presents a flowchart of skeletal survey samples and incidences of patients with a high suspicion of CPA before and during the pandemic.

**TABLE 1:** A comparison of the demographic and injury characteristics of the patients with a high suspicion of child physical abuse before and during the COVID‐19 pandemic.

Characteristic	Before pandemic *N* = 32^a^	During pandemic *N* = 20^a^	*p*‐value^b^
Patient age (days)	249 (160, 388)	132 (56, 389)	0.092
Gender			0.7
Female	11 (34%)	6 (30%)	
Male	21 (66%)	14 (70%)	
Ethnicity			> 0.9
African American	8 (25%)	4 (20%)	
Hispanic	20 (63%)	13 (65%)	
Other	4 (12%)	3 (15%)	
Injury site/type			
Retinal hemorrhage	12 (38%)	1 (5%)	0.008
Bruising	2 (6%)	2 (10%)	0.6
Fracture (nonskull)	15 (47%)	12 (60%)	0.5
Skull fracture	9 (28%)	7 (35%)	0.6
Intracranial hemorrhage and/or infarct	19 (59%)	5 (25%)	0.016
Rib fracture	8 (25%)	3 (15%)	0.5
Upper extremity fracture	8 (25%)	5 (25%)	> 0.9
Lower extremity fracture	6 (19%)	4 (20%)	> 0.9
Clavicular fracture	2 (6%)	5 (25%)	0.092
Spinal fracture	2 (6%)	0 (0%)	0.5
Mandibular fracture	1 (3%)	0 (0%)	> 0.9
Mortality	3 (9%)	0 (0%)	0.28

^a^ Median (Interquartile range, [IQR]); *n* (%).

^b^ Wilcoxon rank sum test; Pearson's chi‐squared test; and Fisher's exact test.

**FIGURE 2:**
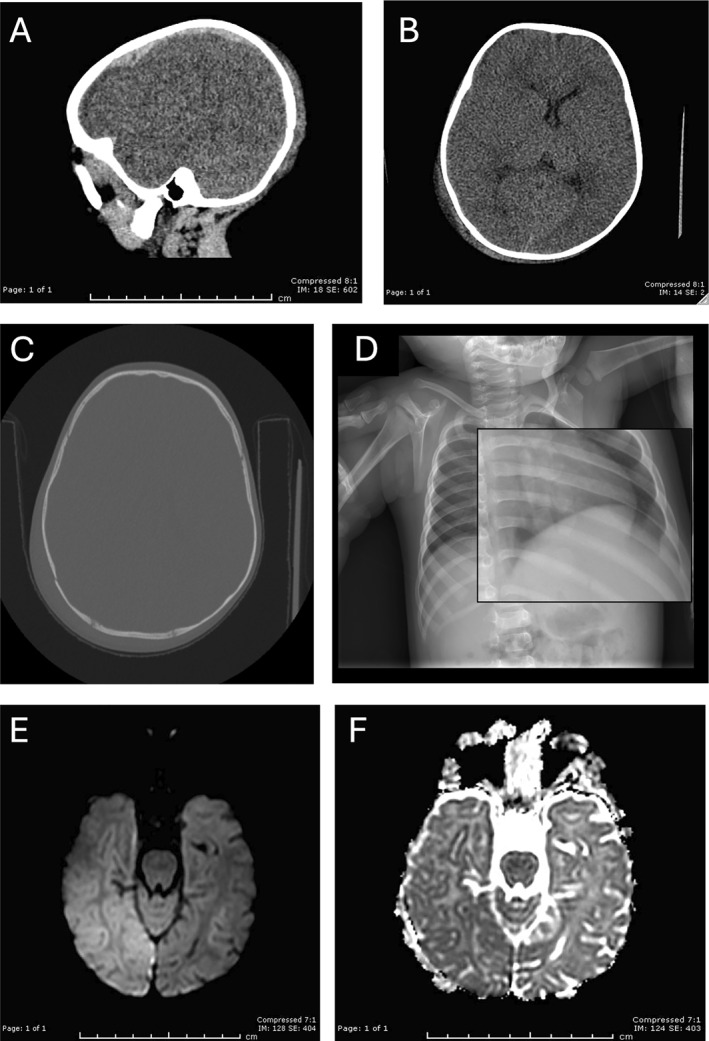
An 8‐month‐old male with a reported unwitnessed fall and bilateral retinal hemorrhages who presented before the pandemic. (A–C) CT axial images demonstrate an acute subdural hematoma with extension along the falx, left to right midline shift, and left‐sided cerebral edema in association with a calvarial fracture involving the right parietal bone with overlying scalp hematoma, (D) the skeletal survey demonstrates periosteal reaction along the left seventh rib posteriorly consistent with healing fracture, and (E and F) MRI diffusion‐weighted images demonstrate infarction of the right cerebral hemisphere.

**FIGURE 3:**
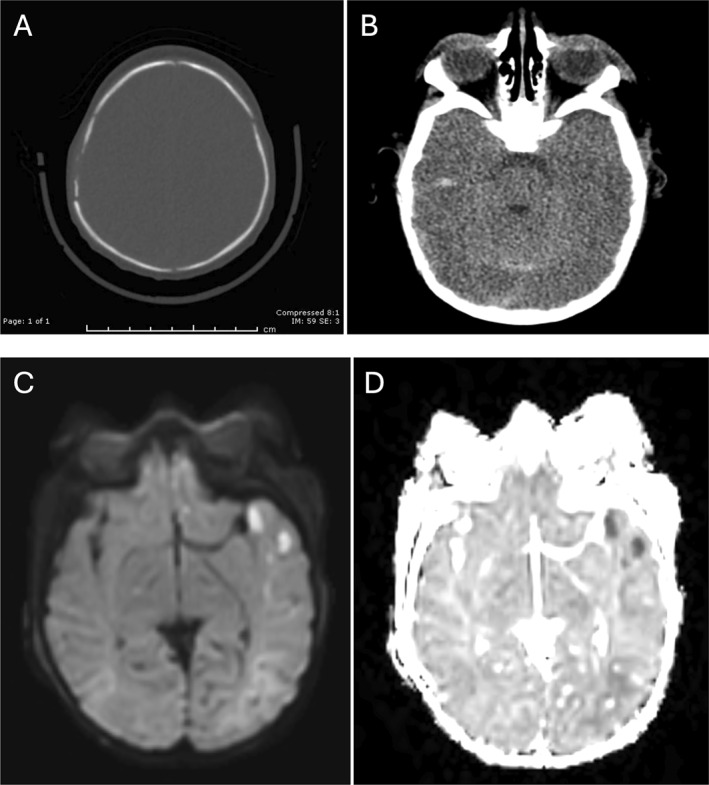
A 5‐week‐old male presented with no known history of trauma who presented during the pandemic with seizure activity and was found to be a victim of unexplained polytrauma, including: (A and B) CT images demonstrating complex right parietal bone fracture, multicompartmental hemorrhage, and cerebral edema and (C and D) MRI demonstrates extensive intracranial injury with multiple areas of restricted diffusion reflecting a combination of contusions, diffuse axonal injury, and watershed infarcts.

**FIGURE 4:**
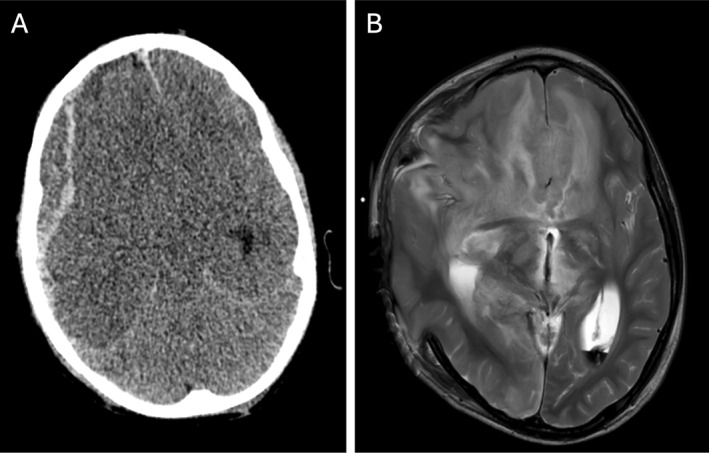
A 6‐year‐old male with significant bilateral retinal hemorrhages, skull fractures, and eventual brain death who presented before the pandemic. (A and B) CT and MRI demonstrate right‐sided subdural hematoma, leftward midline shift, transtentorial/uncal herniation, and infarcts of the entire right cerebral hemisphere and to a lesser degree the left cerebral hemisphere.

**FIGURE 5:**
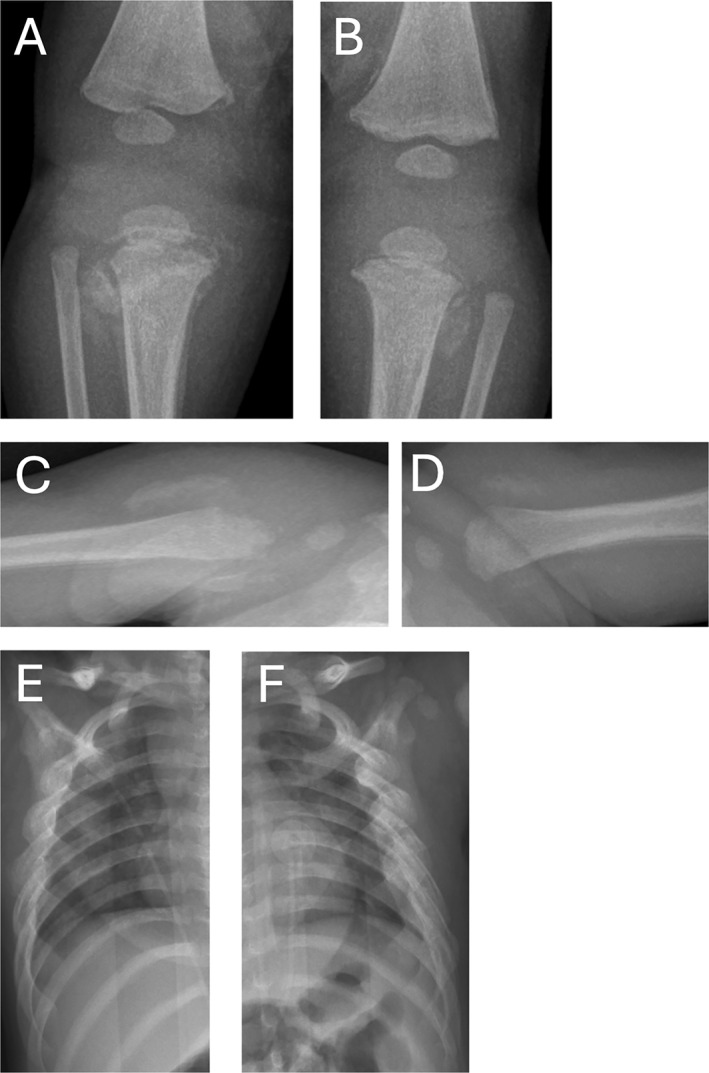
An 81‐day‐old male with witnessed reported physical abuse who presented before the pandemic. (A–D) Multiple fractures in varying stages of healing with radiographic features, such as corner and bucket handle metaphyseal fractures, and (E–F) bilateral clavicular fractures and multiple healing rib fractures.

Representative images for four sample cases of various CPA injuries before and during the COVID‐19 pandemic are shown in Figures 2, 3, 4, 5.

## 4 Discussion

The incidences of cases with a high level of suspicion for CPA detected among children with skeletal surveys were similar before versus during the pandemic comprising 10.5% and 11.6% of all patients who underwent skeletal surveys, respectively. Notably, of those CPA cases, the proportion of retinal hemorrhages (38%) or intracranial hemorrhage (59%) that resulted before the pandemic was significantly higher than the proportion of retinal hemorrhages (5%) and intracranial hemorrhage (25%) that occurred during the pandemic (*p*‐value < 0.05). There was only a single patient with evidence of retinal hemorrhages during the pandemic (Figure 3). All CPA patients with retinal hemorrhages had concomitant intracranial hemorrhage. It has been reported that the severity of retinal hemorrhages is linked with the severity of the acute neurologic injury as well as the presence and size of brain hemorrhage. The most severe cases of CPA among our sample include three patients who incurred abusive head trauma resulting in death. All three patients sustained either bilateral or multilayered bilateral retinal hemorrhages and presented before the pandemic. All other injuries (including skeletal fractures and bruising) as well as demographics revealed no significant (*p*‐value > 0.05) differences before versus during the pandemic as shown in Table 1.

The contributing factors for the decrease of patients presenting with CAN‐related abusive head trauma resulting in retinal hemorrhages and/or intracranial hemorrhage during the pandemic may include (1) decreased monitoring by educators due to school and daycare closures (based on data from the National Child Abuse and Neglect Data System, educators and childcare professionals made 21% and 0.7% reports of CAN in 2019) [2]; (2) an increased hesitancy to visit hospitals and clinics; (3) in‐person visits replaced by telemedicine for many medical professionals at the height of the pandemic, with usual warning signs of child abuse and neglect potentially being missed by providers; and (4) the potential presence of multiple adults at home due to the stay‐at‐home restrictions during the pandemic makes abuse less likely to take place with a protective caregiver and/or surrounding witnesses.

As stated earlier, stressors augmented from the COVID‐19 pandemic may increase the risk for CPA. However, the specific mechanisms by which these factors contribute to an increased CPA risk warrant further discussion. One potential pathway involves the diminished role of schools and childcare centers as frontline detectors of abuse. With school closures, children have less contact with teachers and other mandated reporters, who are often the first to notice signs of abuse. As a result, abuse may go unreported for extended periods, allowing it to escalate until it reaches a severity that necessitates medical intervention, such as hospitalization. In addition to this gap in monitoring, the pandemic exacerbates family stress through economic hardship, social isolation, and the burden of caregiving, potentially leading to increased frustration or negative coping mechanisms, such as substance abuse. These stressors may reduce parents' capacity to manage anger or aggression, thereby increasing the risk of abusive behaviors. By framing these dynamics within a social‐ecological model, which considers individual, relational, community, and societal factors, a clearer understanding of how crises, such as the COVID‐19 pandemic, can amplify CPA risk emerges. This theoretical framework helps to contextualize how reduced external oversight, coupled with intensified household stress, creates conditions conducive to child abuse.

The existing literature on child abuse during the COVID‐19 pandemic presents valuable yet varied insights, and a more critical evaluation is warranted to strengthen the discussion. Several studies provide timely data on increased child abuse injuries, emergency department visits, and the overall well‐being of children and parents during the pandemic, offering crucial perspectives for policymakers and healthcare providers. However, many studies suffer from methodological limitations that complicate the interpretation of findings. For instance, several are based on single‐center or nonrepresentative datasets, limiting their generalizability to broader populations. Cross‐sectional designs, common across these studies, restrict the ability to establish causality, and the reliance on retrospective data often hinders the precise identification of pandemic‐specific effects. Self‐reported measures further introduce biases, potentially underestimating or overestimating the true incidence of abuse. Conflicting findings also arise with some studies reporting a decrease in reported abuse during the pandemic, likely influenced by underreporting due to reduced contact with mandated reporters, whereas others indicate a rise in severe abuse cases reaching healthcare settings. Addressing these discrepancies and expanding longitudinal multicenter research would provide a more comprehensive understanding of the pandemic's impact on child abuse and help clarify conflicting trends across studies.

There are many documented methods that demonstrate reduced risk of CPA. It has been shown that a strong emotional support network is associated with fewer depressive symptoms, especially necessary in a situation as undeniably stressful as the COVID‐19 pandemic [17]. Social support can also be used to counteract the feeling of isolation and anxiety; online peer support groups, resources for those struggling financially, and mental health services can contribute toward parent well‐being. For mandatory reporters, such as teachers or doctors, increased education and awareness of hallmark signs of CPA, especially signs detectable through online platforms, such as unusual personality changes, facial bruising, or inconsistencies, in stories between caregiver and child can be used as clues to suspect potential CPA.

Notable limitations of the study and study design include its retrospective nature. There was not always enough evidence to disqualify confounding variables that might have influenced the CPA assessment, and demographic data were not stringently collected. In addition, there may be some subjectivity and uncertainty with the CPA determination as it was primarily based on documentation from clinicians and social work teams during a patient's admission, without review of additional and/or subsequent reports and follow‐up by investigating agencies and/or the hospital child protection team. The numbers of CPA cases, especially the more significant injuries that were identified, were relatively low, preventing us from performing a comprehensive statistical analysis. The sampling frame of the study was also based on all patients who underwent skeletal surveys for possible child abuse at our specific academic institution, which may not be generalized for the entire population. Future studies would be designed to address these limitations, including, for example, comparing CPA incidences and types of injuries before the pandemic, during the pandemic restrictions, and after schools opened and examining the different types of abusive head trauma and other injuries aside from intracranial hemorrhage. Repeating the study in different geographical regions could also be informational for the purpose of generalizing the results from this study to other healthcare settings as well.

Although the incidences of child physical abuse detectable via skeletal surveys were similar before and during the COVID‐19 pandemic, the incidences of abusive head injuries with retinal hemorrhages, intracranial hematomas, and/or infarcts in our sample significantly decreased during the pandemic. Future studies with more comprehensive analysis on larger datasets, including the data after schools opened, and different types of abusive injuries are required.

## Author Contribution

V.L. and A.S. collected and analyzed the imaging data. V.L. and J.M.R. wrote the first draft of the manuscript. All authors contributed to the design of the study and editing of the manuscript. All authors read and approved the final manuscript.

## Ethics Statement

This study was approved by the Institutional Review Board (IRB) of the Children's Hospital Los Angeles, approval number CHLA‐21‐00210. Since the study was retrospective and deemed exempt, no patient consent was required.

## Conflicts of Interest

The authors declare no conflicts of interest.

## Permission to Reproduce Material from Other Sources

The authors have nothing to report.

## Data Availability

The authors have nothing to report.
